# Drug–Drug Conjugates as Intelligent Preclinical Platforms for Targeted Cancer Therapy

**DOI:** 10.3390/biom16101417

**Published:** 2026-09-30

**Authors:** Aya Abufara, Mohamad A. K. Mousa, Hamdi Nsairat

**Affiliations:** Pharmacological and Diagnostic Research Center, Faculty of Pharmacy, Al-Ahliyya Amman University, Amman 19328, Jordan; ayataher323@gmail.com (A.A.); m.mousa@ammanu.edu.jo (M.A.K.M.)

**Keywords:** drug–drug conjugates, mutual prodrugs, stimulus-responsive release, self-immolative spacers, targeted cancer therapy

## Abstract

Drug–drug conjugates (DDCs), in which two active pharmaceutical ingredients (APIs) are linked covalently within a single molecular structure to produce a synergistic therapeutic effect, represent an emerging category of single-molecule therapeutics. These conjugates differ from conventional combination therapies by utilising well-defined covalent chemical linkages (such as amide, ester, disulfide, and carbamate bonds), usually coupled with self-immolative spacers. This structural architecture ensures synchronised intracellular delivery and controlled, stimulus-responsive release of the parent APIs in a fixed stoichiometry. In oncology, this strategy is especially attractive because it reduces systemic toxicity, improves intratumoral accumulation, and helps bypass resistance mechanisms that limit conventional chemotherapy. Recent advances in linker chemistry, tumour-responsive cleavage, and self-immolative systems have shifted these constructs from simple mutual prodrugs toward intelligent, adaptive delivery platforms. This review summarizes how DDCs mitigate off-target effects, overcome cellular resistance, exploit tumor-specific triggers, and evolve into next-generation translational cancer therapeutics.

## 1. Introduction

Conventional combination chemotherapy is often limited by incompatible pharmacokinetics, variable tissue distribution, and dose-limiting toxicity, which can weaken synergy and increase side effects [1,2]. Beyond standard combination chemotherapy, various nanocarriers—including polymeric systems, albumin-based nanoparticles, and hybrid nanocomposites—have been engineered to co-deliver two therapeutic agents simultaneously [3,4,5,6]. While these platforms help maintain drug ratios, improve tumor accumulation, and modulate multidrug resistance, they typically rely on physical co-encapsulation, adsorption, or independent association rather than true chemical integration [7,8,9]. This limitation highlights the distinct advantage of covalent drug–drug conjugates (DDCs), which offer a reliable way to achieve fixed stoichiometry and chemically programmed dual-drug activation. DDCs address these limitations by chemically joining two active drugs into one construct, enabling fixed stoichiometry and coordinated release [10,11].

Polymeric and hydrogel-based systems may also accommodate two drugs within the same construct, either through physical co-encapsulation or by covalent attachment of the drugs to a common polymeric scaffold. In such systems, the polymer serves as the carrier or structural backbone, whereas in a DDC the two pharmacologically active agents themselves are covalently connected through a defined linker [12,13,14].

DDCs differ from other types of conjugates such as Small-Molecule Drug Conjugates (SMDCs) or Antibody Drug Conjugates (ADCs), whose main function is directly delivering a single cytotoxic drug through a targeting ligand [15]. Specifically, ADCs consist of a monoclonal antibody that recognises a tumour-associated antigen, a cleavable or non-cleavable linker, and a highly potent cytotoxic payload; targeting is achieved via high-affinity antigen binding, followed by receptor-mediated endocytosis and intracellular payload release in endo-lysosomal compartments [16]. SMDCs follow an analogous “targeting moiety–linker–payload” architecture but replace the antibody with a low-molecular-weight ligand (e.g., peptide, vitamin, or receptor binder), which can improve tumour penetration and simplify synthesis while still delivering a single cytotoxic agent to antigen- or receptor-overexpressing cells [17].

In contrast, both components of DDCs are pharmacologically active or pharmacomodulatory [18]. Thus, whereas ADCs and SMDCs are fundamentally single-payload, ligand-targeted delivery systems, DDCs are dual-activity prodrugs in which neither moiety serves primarily as a targeting ligand; instead, tumour selectivity arises from conditional activation of the covalent bond linking the two drugs [19]. Moreover, the bond linking these two active pharmaceutical ingredients (APIs) is engineered as a self-targeting prodrug, triggering full activation under specific cellular conditions, such as enzymatic activity, pH shifts, or redox changes [20,21,22]. Furthermore, recent advances in linker chemistry—specifically self-immolative scaffolds—allow both parent APIs to be released in their native, unaltered states [23,24]. In cancer therapy, this approach is particularly important because it allows the delivery of a potent cytotoxic agent together with a sensitiser, modulator, or second therapeutic partner while maintaining a more controlled exposure profile [25]. Consequently, DDCs emphasise coordinated dual-drug pharmacology and stimulus-responsive release rather than ligand-driven selective delivery of a single payload, distinguishing them mechanistically and design-wise from both ADCs and SMDCs [26]. Reviews on tumour-targeted conjugates emphasise that covalent linkage and selective release are central to improving therapeutic index and minimising adverse effects [27,28].

## 2. The Mutual Prodrug Concept: Beyond Simple Combinations

**Figure 1:**
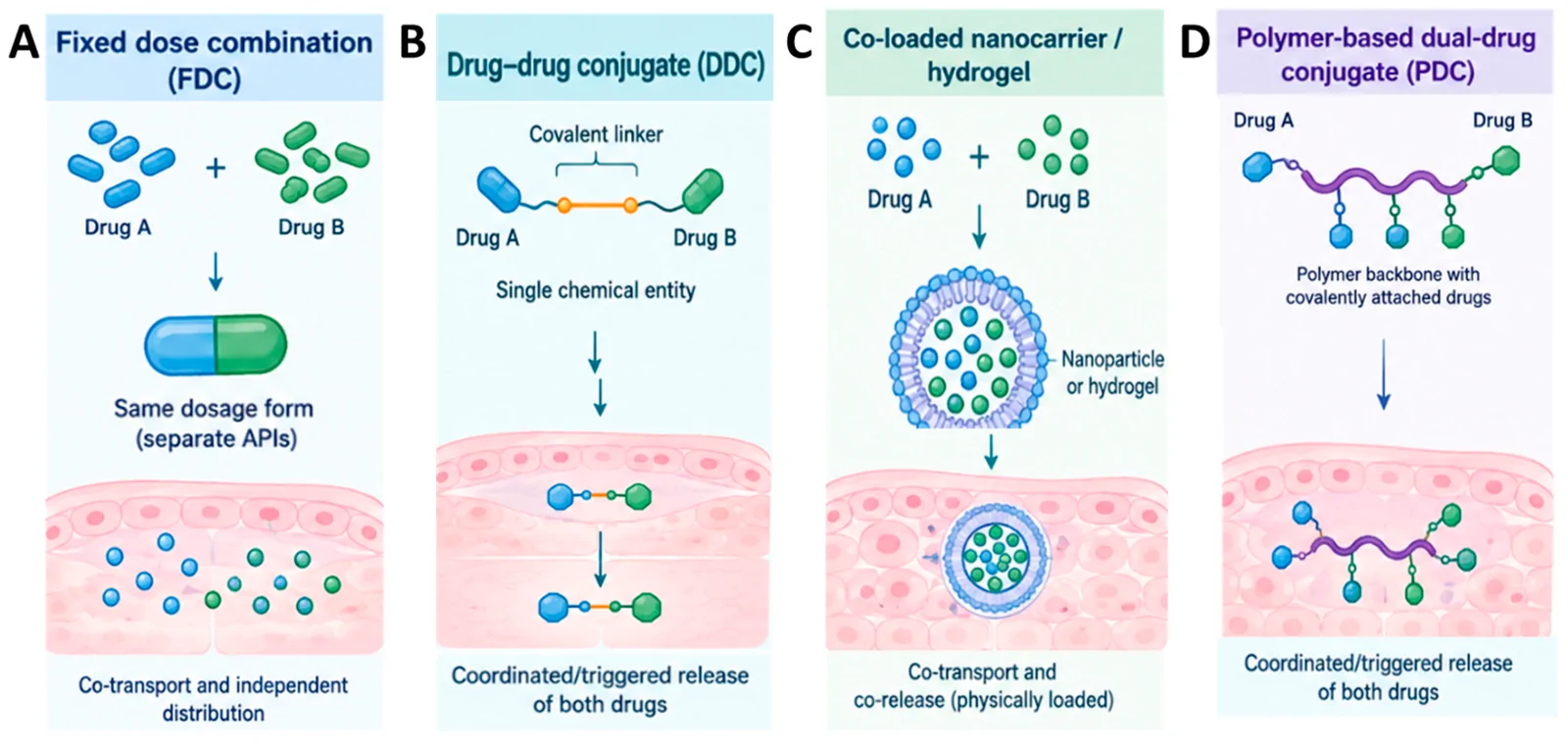
Schematic comparison of multi-drug delivery strategies illustrating their structural frameworks and cellular release dynamics. The illustration outlines four distinct approaches: (**A**) The Fixed Dose Combination (FDC), which packages separate active pharmaceutical ingredients into one dosage form leading to independent intracellular distribution; (**B**) The Drug–Drug Conjugate (DDC), which links two drugs via a covalent bond into a single chemical entity for coordinated intracellular release; (**C**) The Co-loaded Nanocarrier/Hydrogel, which physically encapsulates multiple agents for synchronized co-transport and co-release; and (**D**) The Polymer-based Dual-Drug Conjugate (PDC), which utilizes a polymer backbone functionalized with covalently attached therapeutic agents to ensure precise, coordinated release kinetics.

DDCs consist of two APIs linked together by a covalent bond or via a linker to form a single chemical structure; each API acts as a pro-moiety for the other in a mutual prodrug design [29]. In contrast to fixed dose combinations (FDCs), where each API coexists in the same capsule as a distinct entity, non-bonded [30] (Figure 1). A classic and clinical example of approved mutual prodrugs by the FDA is estramustine phosphate sodium, where a nitrogen-mustard alkylator is linked to estradiol-derived antiandrogen via a carbamate bond [31,32]. Sultamicillin is another example. where ampicillin is linked to sulbactam, which is a β-lactamase inhibitor, through an ester linkage. Upon enzymatic hydrolysis of the ester bond, both APIs are released in a synchronised manner [33,34]. These early examples clarify how a simple covalent linkage between two APIs can turn two distinct pharmacophores into a single structure that behaves differently to enhance either absorption, distribution, or release kinetics, while conserving the intrinsic mechanism of action of the original drugs [35].

This distinction is particularly relevant to polymer-based combination systems, in which two drugs may be covalently incorporated into the same polymeric construct and consequently transported together to tissues or cells. Although such systems can provide coordinated delivery and stimulus-responsive release, the drugs remain independently linked to the polymer backbone rather than being covalently linked to one another as in a DDC [36,37]. Thus, polymer-based dual-drug systems and DDCs share the potential for controlled co-delivery, but differ in their molecular architecture: the former uses a polymer as the common carrier/scaffold, whereas the latter integrates the two APIs into a single drug–drug chemical entity [38].

A schematic comparison of multi-drug delivery strategies illustrating their structural frameworks and cellular release dynamics has been illustrated in Figure 1. The illustration outlines four distinct approaches: the Fixed Dose Combination (FDC), which packages separate active pharmaceutical ingredients into a single dosage form leading to independent intracellular distribution; the Drug–Drug Conjugate (DDC), which links two drugs via a covalent linker into a single chemical entity for coordinated or triggered intracellular release; the Co-loaded Nanocarrier/Hydrogel, which physically encapsulates multiple therapeutic agents within a nanoparticle or hydrogel matrix for synchronized co-transport and co-release; and the Polymer-based Dual-Drug Conjugate (PDC), which utilizes a polymer backbone functionalized with covalently attached drugs to ensure precise, coordinated release kinetics for both agents [39,40,41].

## 3. Architecture of the Hybrid Molecule

There are two ways to form DDCs: either through direct covalent linkage (e.g., ester, amide, carbamate) or via a chemical spacer tailored to control both release kinetics and physicochemical properties [42,43,44]. In spacer-linked systems, the linker is designed to stay stable in the blood but breaks down at the tumour site. This target-specific release is triggered by factors in the tumour microenvironment, such as high glutathione levels, low pH, or overexpressed enzymes like cathepsin B and β-glucuronidase [45,46,47].

These smart delivery systems are categorized into enzyme-sensitive, pH-sensitive, and redox-sensitive architectures based on their primary trigger: enzyme-sensitive linkers use peptide sequences cleaved by intracellular or tumor-associated proteases like cathepsin B, pH-sensitive linkages utilize acid-labile groups such as imines, hydrazones, and acetals that break down in acidic tumor microenvironments or endo-lysosomal compartments, and redox-sensitive systems rely on disulfide or reducible bonds that remain stable in circulation but cleave under intracellular reducing conditions found in glutathione-rich compartments [48,49,50].

Importantly, the distinction between direct and spacer-mediated DDCs refers to the molecular architecture rather than to the type of covalent bond itself. In a direct DDC, the two drug molecules are connected directly through a covalent bond formed between functional groups present on the respective drugs, with no additional molecular segment between them [51]. In contrast, a linker- or spacer-mediated DDC contains an additional chemical unit positioned between Drug A and Drug B; the linker may incorporate one or more covalent bonds that determine its stability and cleavage behavior. Thus, ester, amide, carbamate, hydrazone, or disulfide chemistry can occur either as the direct drug–drug connection or as part of a longer linker architecture. Linkers may additionally be engineered to modify molecular spacing, hydrophilicity, flexibility, stability, and release behavior, including the incorporation of cleavable or self-immolative elements that facilitate release of the parent drugs after exposure to the appropriate biological trigger [52].

**Figure 2:**
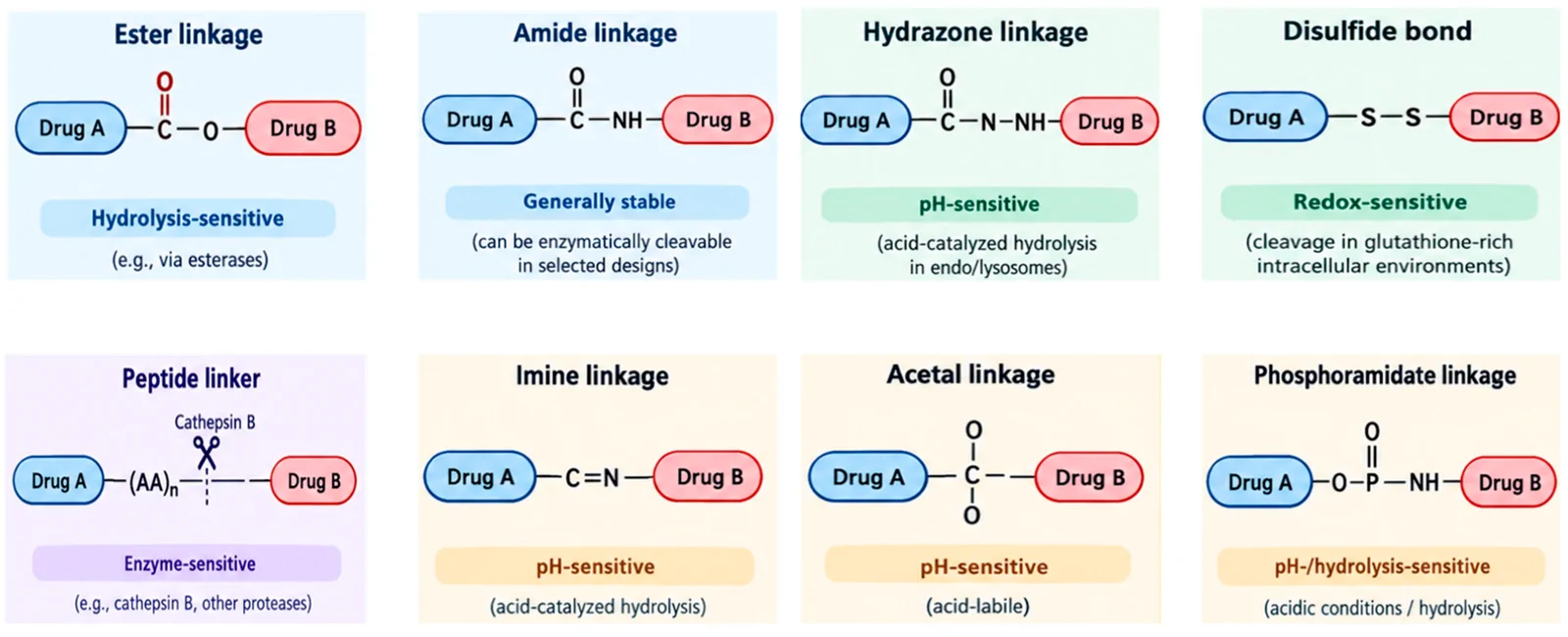
Representative covalent linkages and stimulus-responsive architectures used in drug–drug conjugates (DDCs). Linkages can be designed to undergo enzymatic, pH-dependent, redox-sensitive, or hydrolytic cleavage, enabling controlled release of the conjugated drugs in tumor-associated or intracellular environments.

Common cleavable linkages include esters, amides, carbamates, and disulfides, which connect active drug molecules either directly or through spacers [53] (Figure 2). Each chemical bond within a conjugate has distinct properties that govern its site-specific cleavage. While disulfide bonds are selectively reduced intracellularly within glutathione-rich tumour environments [54], ester and amide linkages undergo enzymatic hydrolysis via localised tissue esterases or amidases [55], and hydrazone bonds function as acid-labile moieties that degrade inside mildly acidic endo-lysosomal compartments [56].

Peptide-based linkers are designed to be cut by specific enzymes inside cells. By placing cathepsin B-cleavable peptide sequences between drug units (or between a drug and a self-immolative spacer), the linker stays intact in the bloodstream but is cleaved after the conjugate enters tumor cells, where protease activity is higher. This enzyme-triggered breakage starts linker fragmentation and releases the active drugs selectively inside the cell [57,58].

pH-sensitive linkers exploit the acidity of tumor microenvironments and intracellular compartments. Bonds such as imines, hydrazones, and acetals break more readily under acidic conditions, while phosphoramidate linkages can be tuned to remain stable at normal physiological pH but hydrolyze in acidic endosomes or lysosomes. This allows the conjugate to stay intact during circulation and release its payload after internalization into acidic compartments [59,60].

Redox-sensitive linkers, such as disulfides, add another control mechanism by responding to the high glutathione levels inside cells. A disulfide-containing conjugate can remain stable in blood but be reduced and cleaved inside glutathione-rich intracellular spaces. Used together, peptide-, pH-, and redox-sensitive linkers provide complementary ways to achieve controlled, site-specific drug release while minimizing premature release in circulation [61,62].

Directly linked DDCs can provide a more compact molecular architecture and may be advantageous when the two drugs possess suitably positioned functional groups, whereas spacer-containing DDCs provide greater flexibility for independently tuning drug separation, physicochemical properties, and triggered release; consequently, both architectures represent complementary approaches to DDC design [63].

Recently, smart self-immolative linkers (especially para-aminobenzyl alcohol, PABC-type systems) have gained significant attention. Upon reduction, hydrolysis, or enzymatic action, it cleaves rapidly via 1,4- or 1,6-elimination [24]. This process releases both drugs in their original forms without leaving any chemical “linker residues” that could lower their therapeutic effectiveness. Eventually, these smart linkers separate the chemistry of the drugs from the conjugate system, allowing a flexible “plug-and-play” design for many different types of medications [64,65].

Moreover, multi-functional transition metal complexes exploit coordinate covalent or structurally cleavable bonding with electronegative heteroatoms (such as nitrogen, oxygen, or sulfur) on drug molecules acting as electron-donating Lewis bases to modulate stability, release profiles, and overall therapeutic delivery [66,67]. For example, this strategy utilises the axial coordination positions of an octahedral platinum(IV) core in their complexes to covalently conjugate bioactive ligands via biodegradable linkages such as ester or carbamate functional groups [68]. Illustrating this approach, a novel ruthenium(II) complex utilised the coordination of 5-fluorouracil (5-FU) to form [Ru(5-FU)(PPh3)_2_(bipy)]PF6. This transition-metal framework significantly optimizes therapeutic delivery and anti-colorectal cancer efficacy by systematically downregulating the Akt/mTOR signalling pathway [69]. Expanding on metal-based design strategies, cyano-modulated zinc(II)-Schiff base complexes have been successfully engineered to enable lysosome-targeted fluorescence imaging coupled with pH-responsive camptothecin delivery [70].

Furthermore, DDCs offer an alternative tool to form self-assembling nanocarriers or carrier-free architectures [71,72,73]. For example, paclitaxel (PTX) and doxorubicin (DOX) were covalently linked into a single heterodimeric prodrug via a redox-responsive thioether linkage. This architecture eliminates the need for inert polymer excipients, thereby enabling precise, stoichiometric self-assembly of uniform nanoaggregates with exceptionally high drug-loading capacity. This approach ensures synchronised pharmacokinetics and precise co-delivery of both payloads to the tumour site [74,75]. Similarly, Zhao et al. demonstrated the therapeutic utility of a synergistic delivery architecture designed for the dual controlled release of camptothecin and doxorubicin, highlighting how coordinated payload liberation enhances combination efficacy [76].

## 4. Bypassing Cellular Defence: Strategies for MDR Reversal

One of the key roles for DDCs is to overcome multidrug resistance (MDR) through conjugation of a cytotoxic agent with a sensitiser or efflux pump inhibitor in a single molecular framework [77,78]. In conventional physical mixtures, the chemotherapy agent is often pumped out of the cell before reaching effective concentrations because its inhibitor partner is cleared more rapidly from the system [79]. In contrast, DDCs ensure that both agents enter the cell at a fixed 1:1 (or 2:1) molar ratio, so that the inhibitor is present at the moment the cytotoxic drug is released [80,81,82].

For example, Cao et al. designed Pt(IV) prodrugs that covalently link a cisplatin-like DNA-damaging core to P-glycoprotein inhibitors, creating a single DDC that reverses resistance in P-gp-overexpressing gastric cancer cells. These conjugates downregulate P-gp, increase intracellular platinum accumulation, enhance DNA damage, and restore apoptosis in cisplatin-resistant, P-gp-high SGC-7901/CDDP cells [68].

Régina et al. designed a DDC in which paclitaxel was conjugated to a brain peptide vector, Angiopep-2, to provide a paclitaxel–Angiopep-2 conjugate (ANG1005). In preclinical studies, ANG1005 entered the brain more efficiently than paclitaxel, bypassed P-gp, retained similar antineoplastic activity in cancer cell lines, showed stronger inhibition of human tumour xenografts, and increased survival in mice with intracerebral glioblastoma or lung carcinoma models [83].

A folate–tubulysin conjugate constructed with an all-D enantiomeric linker configuration was designed to withstand premature systemic degradation while maintaining high fidelity for folate receptor (FR)-overexpressing tumour tissues. Upon selective receptor-mediated endocytosis into FR-positive cancer cells, the intramolecular spacer responds directly to the intracellular microenvironment to release its highly potent tubulysin cargo. This precise trigger mechanism allows EC1456 to achieve an approximate 1000-fold level of tumour-selective specificity in vitro [84].

Importantly, the advantage of the DDC approach over separate administration of the two agents is not limited to co-delivery but also involves preservation of the intended drug ratio and coordinated intracellular action. Compared with separately administered cytotoxic drugs and MDR modulators, covalent conjugation can increase the probability that both components reach the same resistant cell and are released within the same intracellular environment, thereby improving synchronized inhibition of drug efflux and cytotoxic activity [85]. For example, the paclitaxel–Angiopep-2 conjugate ANG1005 provides a representative case in which covalent integration of the therapeutic payload with a transport peptide enhanced brain delivery and enabled activity in intracranial tumor models, whereas conventional paclitaxel is limited by restricted brain penetration and P-gp-mediated efflux [83].

## 5. Reducing Systemic Toxicity

One of the main advantages of DDCs is the reduction in systemic side effects. By keeping the therapeutic payload chemically masked during circulation and releasing it preferentially at the tumour site, these systems can reduce premature exposure of healthy tissues to highly toxic drugs [86]. Chen et al. evaluated diverse payloads, including proteolysis-targeting chimaeras, cytotoxins, and imaging probes, in conjunction with receptor-targeting ligands such as GalNAc and folate. This integrated approach enhances antitumor efficacy and improves systemic safety, thereby expanding the therapeutic window that has constrained DDC development and potentially accelerating their clinical translation [87]. Furthermore, emerging therapeutic modalities such as biomolecular condensates are being harnessed to drive targeted protein degradation for intracellular targets, presenting novel ways to expand payload diversity while minimizing off-target toxicity [88].

A further advantage of this strategy is the potential to increase the therapeutic index by reducing nonspecific exposure while maintaining an effective concentration of the active payload at the intended site. In contrast, separately administered drugs may exhibit different pharmacokinetic profiles, tissue distributions, and clearance rates, which can result in unequal exposure and increased systemic toxicity when both agents are administered at their individually effective doses [89]. Thus, DDCs can integrate dose control, synchronized delivery, transport selectivity, and stimulus-responsive release within a single molecular construct, although the magnitude of these benefits remains dependent on the linker chemistry, targeting strategy, drug combination, and disease model [90].

## 6. Tumour-Specific Triggers Toward Intelligent Platforms

**Figure 3:**
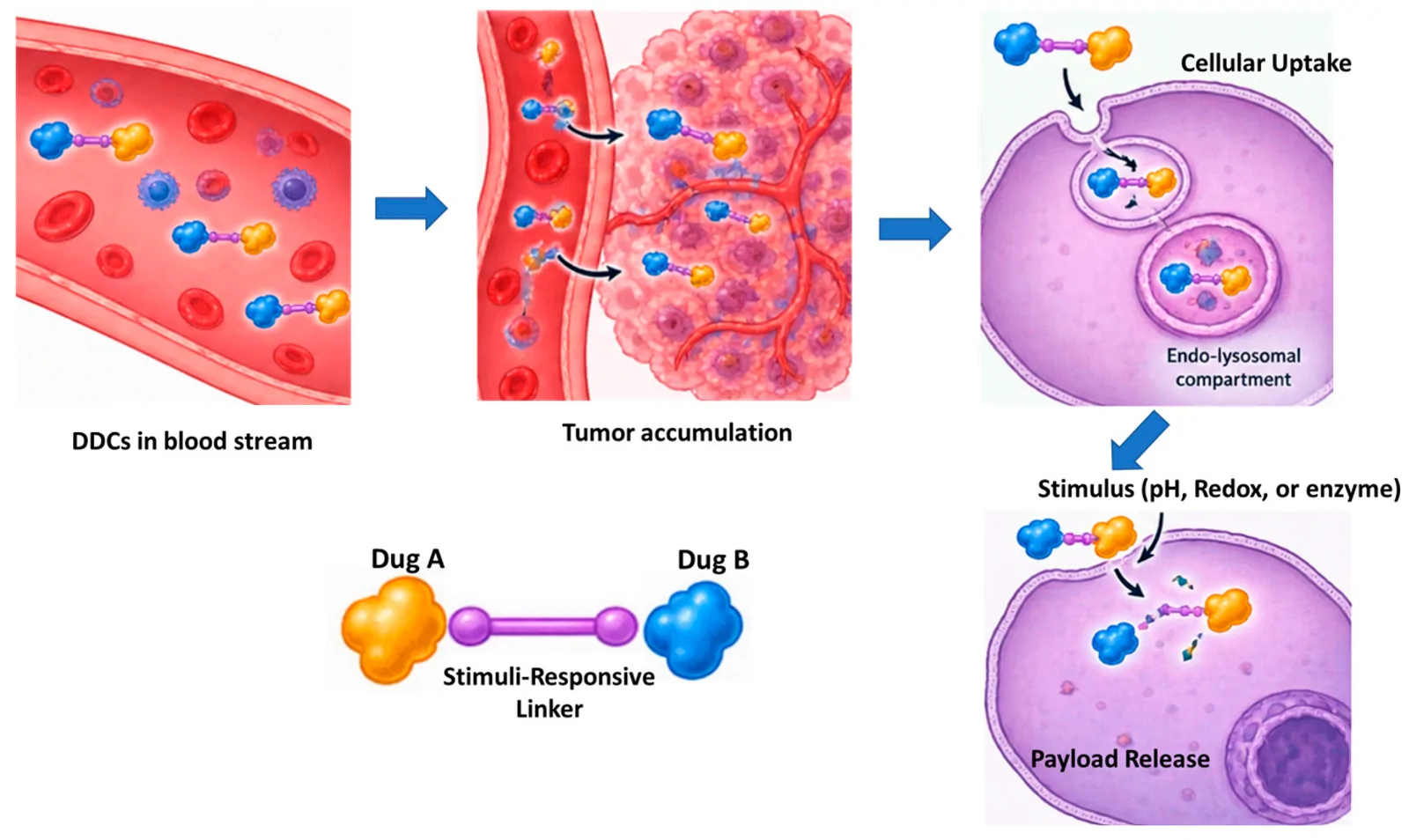
Schematic illustration for the workflow of stimulus-responsive DDCs from systemic circulation to tumor site payload release.

A key advancement in modern DDCs involves the exploitation of tumor-specific triggers to mediate payload release, a dynamic process spanning from stable systemic circulation to targeted intracellular activation, as comprehensively outlined in the workflow schematic (Figure 3). Linkers can be designed to respond to the acidic tumour microenvironment, elevated intracellular glutathione, or tumour-overexpressed enzymes such as cathepsins and glucuronidases [91,92]. Innovative linker strategies have also extended beyond endogenous cues to include external triggers and bioorthogonal release mechanisms, making payload liberation more selective and tunable [93,94]. This trigger-based design is a key reason why DDCs are increasingly viewed as precision therapeutics rather than simple prodrugs [95].

A newly developed bioorthogonal click-and-release platform leverages enamine N-oxides to enable rapid, traceless, and pH-insensitive bioconjugation and dissociation. This minimal-footprint strategy is efficiently driven by diboron reduction, demonstrating great utility for transient protein modifications and cell-targeted DDCs [94]. Extending earlier findings on pH-sensitive phosphoramidate linkers, Backer et al. demonstrated that the tunability of amine-release is directly governed by the pKa of a proximal acidic group using a series of substituted 2-carboxybenzyl phosphoramidates. [96]. A disulfide linker is usually used in DDCs, which remains intact in circulation (low extracellular GSH) but is rapidly reduced, liberating both drugs inside tumour cells where GSH is high [93]. DDCs using cathepsin-cleavable or MMP-cleavable peptides have been reported where cleavage liberates two cytotoxins or a cytotoxin plus a synergistic agent [97,98].

Crucially, responding to a tumor-specific cue does not automatically ensure that a treatment will act or accumulate selectively in cancer cells, since these same cues can appear in healthy tissues and cause unintended side effects. Consequently, combining trigger-released mechanisms with active tumor-targeting strategies is essential for maximizing therapeutic precision [27].

## 7. Clinical Translation

From a regulatory and clinical-translation viewpoint, DDCs are generally expected to be developed as new molecular entities because covalent conjugation generates a new chemical structure with its own pharmacokinetic, metabolic, and toxicity profile, which may differ substantially from those of the parent drugs. Accordingly, the development package should not rely solely on the established clinical experience of the individual APIs [99]. Key clinical-pharmacology requirements include a validated bioanalytical strategy capable of separately quantifying the intact conjugate, each released API, and relevant metabolites; first-in-human dose selection based on the preclinical pharmacokinetics and toxicology of the conjugate and its constituent drugs; and comprehensive dose–exposure–response analyses [100]. Development must also evaluate the impact of hepatic/renal impairment, demographic factors, and concomitant medications on PK, assess drug–drug interaction potential for the released drugs (and possibly the conjugate), and determine the need for dedicated QTc and other safety pharmacology studies based on the payloads’ risks and exposure margins [101]. Together, these considerations ensure that DDCs are developed with a clear understanding of their multi-analyte pharmacology, distinguishing them from simple fixed-dose combinations.

The preclinical-to-clinical pathway for DDCs is still substantially less mature than that of ADCs and other targeted conjugate platforms. Nevertheless, several development programmes illustrate the increasing range of DDC design strategies. One important class comprises conjugates that combine two kinase-directed agents to address pathway cooperation or adaptive resistance. For example, MEK–AKT inhibitor DDCs have been investigated for RAS-mutant cancers, where simultaneous modulation of the MAPK and PI3K–AKT pathways is intended to provide fixed-ratio pathway inhibition within a single molecular entity [102]. A reported programme generated a series of trametinib-containing DDCs with AKT-directed partners and evaluated their chemical, cellular, and in vivo properties, illustrating the transition from rational API pairing to lead optimisation and preclinical efficacy assessment. These candidates are particularly relevant because their translational rationale is based on coordinated pharmacology rather than on antibody- or receptor-mediated payload delivery [102].

Additional preclinical examples include DDCs that combine a cytotoxic or antimetabolite API with a sensitising, resistance-modifying, or pathway-inhibitory partner. The 5-fluorouracil–DCA series and the 5-fluorouracil–HO-1 inhibitor conjugates exemplify this approach: one component provides direct antitumour activity, whereas the second component is intended to modulate tumour-cell survival or stress-response pathways. Similarly, 6-mercaptopurine–methotrexate conjugates demonstrate how two pharmacologically active antimetabolite components can be incorporated into a single covalent architecture. These examples remain preclinical, but they provide useful evidence that DDC design can be extended beyond two conventional cytotoxic drugs to combinations involving a cytotoxic agent and a pharmacological sensitiser [103].

The translational value of these candidates depends not only on in vitro potency but also on whether the conjugate preserves the intended fixed drug ratio after administration. Therefore, preclinical progression should include parallel measurement of intact DDC exposure, cleavage kinetics, released-parent-drug exposure, tissue distribution, and metabolite formation [104]. In vivo efficacy should be compared with equimolar administration of the unconjugated APIs and, where appropriate, with a non-cleavable or inactive-linker control. Such comparisons are necessary to determine whether any therapeutic advantage results from covalent linkage and coordinated release rather than simply from the combined pharmacological activity of the two drugs [105].

The linker is also a major determinant of translational performance. Cleavable ester, carbonate, carbamate, disulfide, or enzyme-responsive linkers may be designed to release the parent APIs under tumour-associated or intracellular conditions, whereas non-cleavable linkers may generate chemically modified metabolites rather than the native drugs. Consequently, candidate selection must balance plasma stability against sufficiently rapid intracellular activation [45,47]. A conjugate that is too labile may release the APIs prematurely and reproduce the toxicity and pharmacokinetic incompatibility of conventional combination therapy, whereas excessive stability may limit intracellular drug liberation and reduce efficacy. Linker optimisation should therefore be supported by plasma-incubation studies, lysosomal or cytosolic stability assays, tumour and healthy-tissue distribution, and quantitative analysis of the intact conjugate and released products [106].

At present, the DDC field contains more preclinical proof-of-concept programmes than clinically tested candidates. This contrasts with ADCs, for which multiple agents have entered late-stage clinical development and several have received regulatory approval. It is therefore important not to describe preclinical DDCs as clinically validated products. Rather, they should be viewed as a developing pipeline in which the principal translational questions concern reproducible synthesis, batch-to-batch control, linker stability, multi-analyte bioanalysis, predictable release, scalable manufacturing, and the relationship between intact-conjugate exposure and pharmacodynamic activity [107,108].

Recent DDC programmes spanning kinase–inhibitor pairs, antimetabolite combinations, cytotoxic–sensitiser combinations, and other dual-active architectures demonstrate the evolution of the platform from conceptual molecular design toward lead optimisation and translational evaluation. Characterising these candidates according to their API combination, linker type, conjugation site, release mechanism, pharmacokinetic behaviour, and development stage provides a framework for comparing how specific structural strategies influence biological activity and clinical readiness [109,110]. A summary of representative preclinical and clinical-stage examples is shown in Table 1, with the development stage and available translational evidence indicated explicitly. 

## 8. Hansen Solubility Parameter-Based Physicochemical Mapping of Representative DDCs

The Hansen Solubility Parameters (HSPs) were used in this work merely as a means of comparison through its three-dimensional interaction parameter. The three interaction parameters include: dispersion interaction (δD), dipole or polar interaction (δP), and hydrogen bond interaction (δH) [121]. HSPs are not considered predictors of solubility, miscibility, biological activity, or behavior within formulations for the purposes of the present investigation. Instead, HSPs are applied in the determination of relative molecular displacements in Hansen space, and for illustration of the influence that covalent linkage and linker structure have on the contributions to calculated interactions [122,123].

The Hansen total solubility parameter was calculated as follows:

δT = √(δD^2^ + δP^2^ + δH^2^)

Parent compounds and conjugates were analyzed with HSPiP. The neutral molecular structure was input, and HSP values were calculated based on the Y-MB molecular fragmentation method [124]. The reason why neutral structures were used is that salts and counter ions are not within the scope of prediction by the Y-MB method. Notably, the Y-MB approach gives rise to a complete-molecule, fragment-based assessment; hence, the output HSP values should be considered as a relative molecular descriptor and not an experimental material property [125].

The physicochemical distance between two compounds in Hansen space was calculated using the Hansen distance, Ra:

Ra = √[4(δD_1_ − δD_2_)^2^ + (δP_1_ − δP_2_)^2^ + (δH_1_ − δH_2_)^2^]

The lower the Ra value, the closer the two molecules will appear to be separated in the Hansen Space. Yet the Ra value is insufficient to determine whether the molecules are miscible, soluble, or compatible with each other. This information usually requires the determination of the interaction radius (R0) experimentally [126,127]. Since no R0 values could be found for the DDCs considered herein, Ra was employed solely as a relative molecular distance descriptor. In this respect, all references to proximity and/or displacement herein pertain to those computed in Hansen space.

The analysis reveals that covalent binding is not just creating an average of the parent HSPs (Table 2) [128]. The separation in Ra between the parent APIs for the 6-mercaptopurine–methotrexate combination was 11.28 MPa^1/2^. However, when the conjugate was evaluated against 6-mercaptopurine, the Ra was 10.40 MPa^1/2^, while when compared with methotrexate, the value was 4.76 MPa^1/2^. [129]. Hence, in terms of the descriptor space used, the conjugate is more similar to methotrexate. The major numerical similarities between the two drugs are in δD and δP (20.6 and 13.3 MPa^1/2^ for the conjugate and 20.5 and 12.1 MPa^1/2^ for methotrexate). However, δH for the conjugate decreases to 11.8 MPa^1/2^ [130].

Asymmetry is even more pronounced in the 5-fluorouracil–HO-1 inhibitor complex. Separation between the parent molecules was 12.80 MPa^1/2^, but that between the conjugated molecules was 4.52 MPa^1/2^ in case of imidazole HO-1 inhibitor and 13.72 MPa^1/2^ in case of 5-fluorouracil. In the conjugated complex, both δP and δH have the same values (δP is 8.0 MPa^1/2^; δH is 6.2 MPa^1/2^). Compared to 5-fluorouracil, both parameters decrease [131].

From a mechanistic perspective, the calculated shift could be attributed to the formation of a different covalent arrangement by the linker through the conversion of functional groups: ester/linker formation affects the number and type of polar and hydrogen bonding functional groups described by the group contribution model, whereas the hydrocarbon/dispersion contribution of the entire scaffold is not significantly affected. The discussion here is about the calculated interaction profile and not about the experimental solubility or permeability effects. The 5-fluorouracil–DCA family shows the most unambiguous examples of linker-induced HSP shift. The parent drug compounds showed an Ra difference of 11.28 MPa^1/2^. For compound 1a, the Ra values compared to 5-fluorouracil and DCA were 8.18 and 6.52 MPa^1/2^, while for compound 1c they were 11.90 and 5.21 MPa^1/2^, respectively. The two conjugated compounds showed an Ra difference of 4.04 MP.

While their δD values stayed almost identical (18.5 and 18.3 MPa^1/2^ respectively), δP went down from 13.0 for 1a to 9.0 MPa^1/2^ for 1c, while δH dropped only marginally, from 8.6 to 8.2 MPa^1/2^. It thus becomes clear that altering the nature of the linker in compounds belonging to the same API pair affected the polar contribution significantly, while leaving the dispersion one virtually unchanged. This means that the linker itself has the ability to modify the contribution of polar fragments versus non-polar ones through changes in molecular weight and structure. The shift in the profile of 1c away from that of 5-fluorouracil should thus be considered the result of such modification, and not the inherent lipophilicity or solubility of the latter.

Overall, in all three systems, δD was less affected by conjugation compared to δP and δH, which implies that the most apparent impact of conjugation in our data set is the re-allocation of polar and hydrogen bond donor/acceptor contributions. The influence of the structure of a linker on these terms is possible in several ways: (i) esterification, amidation, carbamatisation or disulfide formation might lead to the deactivation or transformation of donors/acceptors present in the original compounds; (ii) the aliphaticity of the linker increases the contribution of dispersion-dominated terms; and (iii) the presence of heteroatoms, carbonyl group or ionisable groups might increase or redistribute the polar and hydrogen bond donor/acceptor contributions. Thus, different linkers and conjugation sites may cause different changes in the position of the DDC in Hansen space using the same two APIs. Since these values are average whole-molecule terms, their changes should be treated as mechanistic descriptors of the structural change rather than solubility/biological data [132].

### HSP Analysis Scope in the Development of DDC Based on Linker Interpretation

In spite of all these restrictions, however, HSP mapping could be a valuable tool to make initial comparisons of structure-related DDCs. The greatest use that can be made out of HSP mapping at the moment would be the comparative one; the variation in δD, δP, δH and Ra values would help to see how the introduction of a certain linker, linker polarity, the location of conjugation, or the deprotection of the group affect the overall interaction potential of the molecule. For instance, the introduction of new aliphatic moieties into the linker would reduce the polar contribution of the molecule while, on the contrary, a more functional linker might raise the values of δP and/or δH [133].

Thus, the computed values in this work must not be considered as prediction for water solubility, permeability through membranes, hydrolytic reactivity, pharmacokinetic profiles, antitumor activity, or synergistic activity for drugs. Also, by limiting only one HSP triplet for each larger DDC, this means that any chemically diverse areas become averaged within a molecule as one unit, which hides local hydrophilicity and hydrophobicity. This work, thus, purposely confines the use of HSP computation to only physicochemical mapping and molecular-distance comparison. Further physicochemical interpretation can only be derived from experimentally determined solubility/miscibility, radius of interaction, chromatography/spectroscopy, and solid state/particle characterization data [134].

## 9. Future Directions: Triple-Drug Hybrids and Smart Release

**Figure 4:**
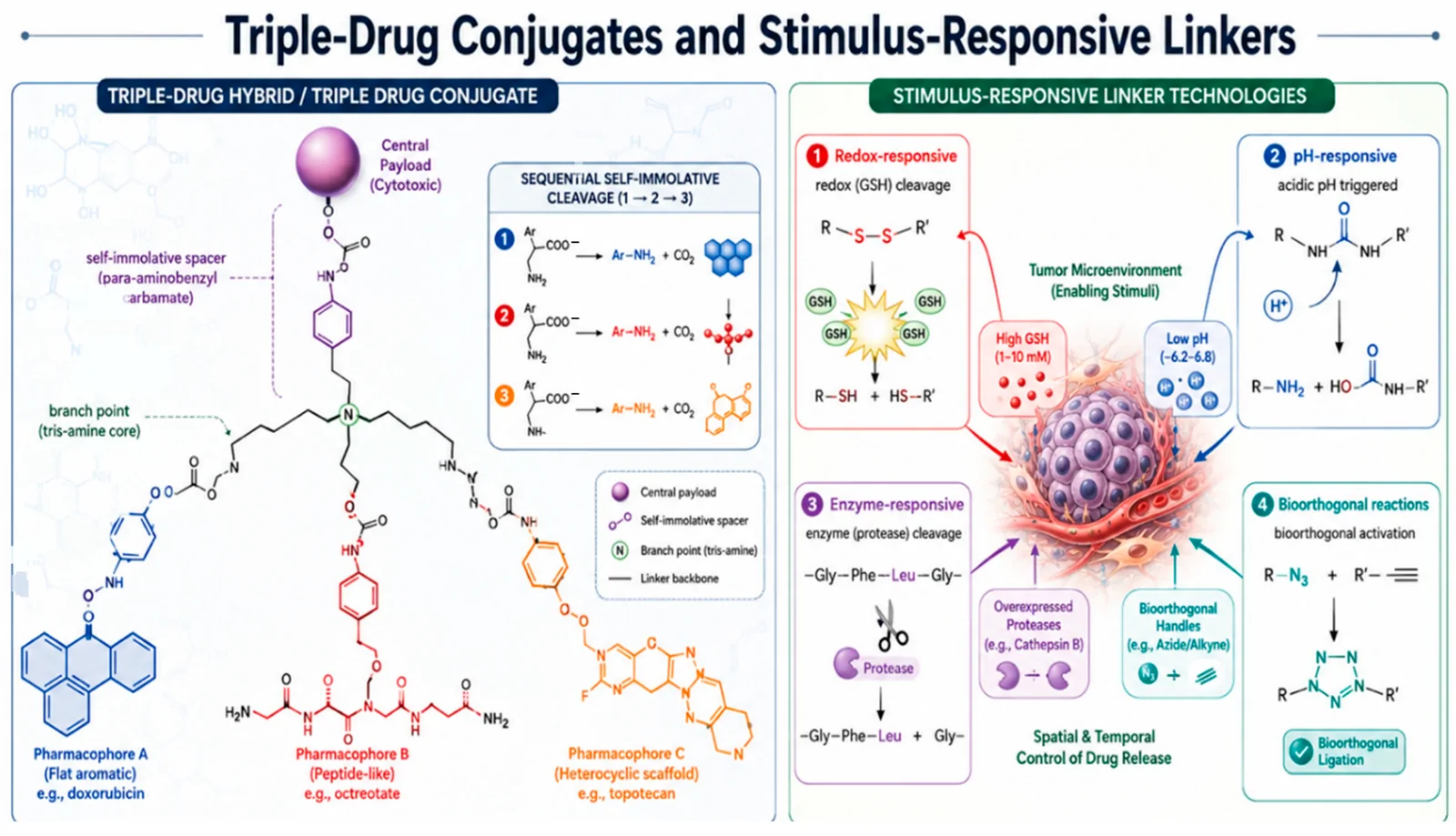
Schematic representation of triple-drug conjugate design and stimulus-responsive linker technologies.

Emerging research explores triple-drug conjugates in which three distinct pharmacophores are tethered via a sequential self-immolative structure or branching linker, enabling simultaneous targeting of multiple pathways (e.g., inflammation, cytotoxicity and immunomodulation mechanisms) within a single molecular framework [135]. At the same time, advances in stimulus-responsive linker technologies such as responsiveness to redox, pH state, enzyme conditions, or even biorthogonal reactions have contributed to the emergence of smart DDC technology [64] (Figure 4).

These trends indicate that API–API conjugate platforms will evolve from simple mutual prodrugs to multi-payload and programmable platforms that are capable of integrating synergy, bypassing resistance, and improving drug convenience by making it a single chemical structure. As synthesis methodology and analytical tools evolve, the field is expected to expand and go from simple dual-drug combinations to mechanism-oriented, rationally designed, tri-therapeutic conjugates system and optimized for specific disease conditions [135,136].

### Synthetic and Pharmacokinetic Bottlenecks

Despite their therapeutic promise, the clinical translation of triple-drug conjugates faces substantial synthetic and pharmacokinetic hurdles; constructing a single molecular entity bearing three distinct functional groups requires multi-step synthetic routes with extensive use of orthogonal protecting groups [137]. This often leads to low overall reaction yields, challenging purification processes, and difficulties in scaling up production. Moreover, integrating three diverse pharmacophores drastically increases molecular weight and often pushes the compound outside of standard drug-likeness parameters (such as Lipinski’s Rule of Five). This can result in severely compromised aqueous solubility, excessive lipophilicity, and aggregation tendencies [138]. Achieving precise, sequential, and stoichiometric release of all three active agents remains difficult. Premature cleavage in systemic circulation can lead to off-target toxicity, while steric hindrance from the branched scaffold can prevent proper target engagement or cellular uptake. Furthermore, altered pharmacokinetic profiles may cause uneven clearance rates among the released components, complicating predictable in vivo behavior [19,139].

Mustafa and Al-Omari reported a coumarin-based triple mutual prodrug for lung cancer reported illustrates the emerging “triple-drug hybrid” concept: three pharmacophores are unified in one scaffold so that, upon biological activation, multiple complementary anti-cancer mechanisms can be delivered from a single molecule. This early design foreshadows current efforts to build smarter, stimulus-responsive tri-therapeutic conjugates that use self-immolative or branching linkers to achieve coordinated, site-selective release and enhanced synergy while simplifying dosing into one rationally optimised chemical entity [140].

Future DDC development will therefore require balancing the potential advantages of multi-payload and smart-release systems with practical limitations such as synthetic complexity, manufacturing cost, scalability, pharmacokinetic control, and potential off-target toxicity [137,141].

## 10. Conclusions

DDC platforms represent a powerful evolution that exceeds traditional combination therapy, converting two separate APIs into a single, self-targeting mutual prodrug with stimulus-responsive activation and synchronised pharmacokinetics. By the activation of disulfide, ester, amide, hydrazone bonds and self-immolative linkers, these conjugates overcome pharmacokinetic inconsistency and multidrug resistance, improving synergy, particularly in complex infections and oncology.

As the field is progressing toward more advanced architectures, including triple-drug hybrid platforms and environmentally responsive release systems, API–API conjugates are arising as a foundational strategy for next-generation therapeutics. Their classification as New Molecular Entities (NMEs) emphasises the need for careful mechanistic and regulatory assessment, but also provides a clear pathway toward clinically relevant, multi-mechanistic therapies that integrate sophisticated molecular engineering with critical therapeutic potential.

## Figures and Tables

**Table 1:** Characteristics and therapeutic evaluations of reported covalent drug–drug conjugates and ligand–targeted constructs across different cancer models.

Drug 1	Drug 2/Targeting Moiety	Linker/Conjugation Chemistry	Release Mechanism/Trigger	Main Outcomes	Development Stage	Ref.
6-Mercaptopurine (6-MP)	Methotrexate (MTX)	Reversible amide/disulfide bond via carbodiimide coupling	GSH/redox and enzymatic cleavage	Enhanced cytotoxicity against human breast (MCF-7) and ovarian (SKO-3) tumor cells.	Preclinical DDC	[111]
5-Fluorouracil (5-FU)	Imidazole-based HO-1 inhibitor	Succinyl ester via EDC/DMAP esterification	Enzymatic ester hydrolysis	Favorable ADME/toxicity profile; selective cytotoxicity against cancer cells (DU145, A549) with reduced normal cell toxicity.	Preclinical DDC	[112]
5-Fluorouracil (5-FU)	Dichloroacetic acid (DCA)	Cleavable ester-based aliphatic linker (chloroacetyl/adipate/succinate)	pH-dependent and enzymatic hydrolysis	Enhanced anticancer activity, apoptosis induction, higher potency, and improved selectivity.	Preclinical DDC	[103].
5-Fluorouracil (5-FU)	Imidazole-based HO-1 inhibitor	Succinyl ester via EDCI/DCC coupling	Esterase-mediated hydrolysis (PBS/porcine serum)	Effective anticancer activity, lower normal-cell toxicity, and superior performance over free drug combinations.	Preclinical DDC	[113]
Paclitaxel	Albumin (carrier protein)	Monobromomaleimide-C-2 linker	Serum stabilization	Serum-stabilized conjugate design with improved in vivo persistence and stability.	Carrier drug conjugate; not a strict DDC	[114]
Modified camptothecin payload (VIP126)	αVβ3 integrin binder	Neutrophil elastase (NE)-cleavable linker	NE in the tumor microenvironment	Improved tumor selectivity, strong cytotoxic activity, and tumor regression in mouse models.	Ligand-targeted SMDC	[115]
Acetazolamide derivative	Cytotoxic drugs (MMAE or PNU-159682)	Dipeptide linker	Extracellular proteolytic cleavage at the tumor site; intracellular drug internalization	Selective accumulation in CAIX-positive renal cell carcinoma and potent antitumor activity in xenografts.	Ligand-targeted SMDC	[116]
SN38, docetaxel, or DM1	iRGD peptide motif	Self-immolative disulfide linker	Intracellular thiols (especially reduced glutathione)	Improved aqueous solubility, strong cytotoxicity, reduced tumor migration/invasion, and suppressed tumor growth in vivo.	Peptide drug conjugate comparator	[117]
DM1 maytansinoid	Zinc dipicolylamine-based phosphatidylserine-targeting ligand (Zn8)	Thioester-linker	Intracellular processing following tumor targeting	Induced tumor regression in HCC1806 triple-negative breast cancer xenografts and increased “hot inflamed tumor” gene signatures.	Ligand-targeted SMDC	[118]
Avutometinib	ZSTK474	PEG-containing covalent linker	Intended controlled release or intracellular processing	Designed for sustained MEK/PI3K pathway inhibition; reported as a preclinical DDC strategy	Preclinical DDC	[102]
Trametinib	AKT inhibitor, including afuresertib or MK2206	Amide/alkylative coupling through aliphatic, oxyethylene, dipeptide, fluorous, furan, or triazine linkers	Linker-dependent intracellular processing; intact conjugate activity was also evaluated	Dual MEK/AKT pathway inhibition, prolonged murine half-life, and antitumour activity in RAS-mutant xenografts	Preclinical DDC lead series	[119]
NUV-1511	Proprietary second active anticancer moiety within a chimeric small molecule	Proprietary covalent DDC chemistry; detailed structure and release mechanism should be reported from the primary development source	Under clinical investigation; intact-conjugate and metabolite pharmacology are being evaluated	Phase 1/2 dose escalation in advanced solid tumours, including HER2-negative breast, prostate, pancreatic, and ovarian cancers	Early clinical DDC	[120]

**Table 2:** HSPiP-estimated Hansen solubility parameters and relative Hansen distances of representative drug–drug conjugates and their parent compounds.

DDC System	Compound	δD	δP	δH	δT	Ra vs. Drug 1	Ra vs. Drug 2	Main Observation	Ref.
6-MP-MTX	6-Mercaptopurine	22.1	22.8	14.8	35.0	—	11.28	Parent API	[111]
	Methotrexate	20.5	12.1	16.4	28.9	11.28	—	Parent API	
	Intact conjugate	20.6	13.3	11.8	27.2	10.40	4.76	Relative HSP distance nearer to methotrexate	
5-FU-HO-1 inhibitor	5-Fluorouracil	18.8	19.8	13.1	30.3	—	12.80	Parent API	[112]
	Imidazole-based HO-1 inhibitor	20.7	8.0	9.9	24.3	12.80	—	Pharmacological partner	
	Intact conjugate	19.4	8.0	6.2	21.9	13.72	4.52	HSP is more proximal to the HO-1 inhibitor	
5-FU-DCA	5-Fluorouracil	18.8	19.8	13.1	30.3	—	11.28	Parent API	[103]
	Dichloroacetic acid	17.8	8.7	13.3	23.9	11.28	—	Parent API	
	Intact conjugate 1a	18.5	13.0	8.6	24.2	8.18	6.52	Position of HSP relative to DCA changed	
	Intact conjugate 1c	18.3	9.0	8.2	22.0	11.90	5.21	Relative positioning of HSP moved closer to DCA	

Note: δD represents the dispersion contribution, δP represents the polar contribution, δH represents the hydrogen bonding contribution, and δT represents the total Hansen solubility parameter (HSP). Ra is the Hansen distance. All values for the HSP and Ra have units of MPa^1/2^. The values of HSP were determined by employing HSPiP software, version [6.2.05], by using the neutral free forms of the parent drugs and their conjugates. Drug 1 and Drug 2 represent the first and second drugs in each DDC system, respectively. Since experimentally known interaction radius (R0) was unavailable, RED calculation could not be performed. Thus, the values of Ra and HSP can be used only to compare the distances between the molecules.

## Data Availability

No new data were created or analyzed in this study. Data sharing is not applicable to this article.
